# Clinicopathological Characteristics and Survival Outcomes of Primary Renal Leiomyosarcoma

**DOI:** 10.3389/fsurg.2021.704221

**Published:** 2021-10-21

**Authors:** Cheng Chen, Xinjie Jiang, Fei Xia, Xudong Chen, Weiguo Wang

**Affiliations:** Department of Urology, Xiangya Changde Hospital, Changde, China

**Keywords:** kidney, leiomyosarcoma, sarcoma, SEER program, prognosis

## Abstract

**Background:** Primary renal leiomyosarcoma (LMS) is an exceedingly rare entity with a poor prognosis. We summarized the clinicopathological characteristics, treatment choice, and survival outcomes of LMS from the Surveillance, Epidemiology, and End Results (SEER) database.

**Methods:** Renal LMS and kidney renal clear cell carcinoma (KIRC) data from 1998 to 2016 were collected from the SEER database. The continuous variables were analyzed using *t*-tests, while the categorical variables were analyzed using Pearson's chi-squared or Fisher's exact tests. Propensity score matching (PSM) was also performed. The cancer-specific survival (CSS) and overall survival (OS) curves were estimated using Kaplan-Meier analyses and compared by log-rank tests. The risk factors for CSS and OS were estimated using univariable and multivariable Cox proportional hazard regression models.

**Results:** A total of 140 patients with renal LMS and 75,401 patients with KIRC were enrolled. These groups differed significantly in sex, race, tumor size, grade, SEER stage, surgery, radiation, and chemotherapy. Renal LMS exhibited poorer CSS and OS compared with KIRC before and after PSM. For renal LMS, the univariate Cox proportional hazard regression model indicated that larger tumor size, higher tumor grade, higher SEER stage, and chemotherapy were risk factors for CSS and OS, while surgery appeared to be a protective factor. However, only tumor grade, SEER stage, and receiving surgery remained independent prognostic factors in the multivariable Cox proportional hazard regression model. In addition, subgroup analyses indicated that surgery remained a protective factor for advanced renal LMS. However, there was no survival benefit for patients receiving chemotherapy.

**Conclusions:** Primary renal LMS is an exceedingly rare entity with distinct clinicopathological features and a poor prognosis. A higher tumor grade and late stage may indicate a poor prognosis. Complete tumor resection remains to be the first treatment choice, while chemotherapy may be a palliative treatment for patients with advanced disease.

## Background

Renal carcinomas are common neoplasms, with over 300,000 patients diagnosed worldwide each year (1). Clear cell carcinoma, papillary carcinoma, and chromophobe carcinoma are the most common solid carcinomas within the kidney, accounting for more than 85% of all renal malignancies (2). Primary renal leiomyosarcoma (LMS) is an exceedingly rare entity that accounts for only 0.12% of all renal malignancies (3). However, renal LMS, accounting for 50–60% of all cases, is the most common pathological subtype of renal sarcoma (4). Because of its rarity, most reports on renal LMS are case studies (5, 6). Few case series have reported the clinicopathological characteristics, potential treatment choice, and survival outcomes of LMS (7–9), which have not been fully characterized.

Kendal et al. reported the largest renal LMS cohort to date and concluded that LMS exhibited relatively favorable survival outcomes compared with clear cell carcinoma (7). However, most studies have reported an extremely poor prognosis for renal LMS (8–10). In addition, the treatment choices remain controversial. Some cases have reported that renal LMS could benefit from chemotherapy (11) and radiation (12), while other cases reported that chemotherapy and radiation did not appear to alter the clinical outcome (13). In this study, we study the largest cohort of patients with primary renal LMS from the Surveillance, Epidemiology, and End Results (SEER) database to summarize the clinicopathological features, treatment choices, and survival outcomes of primary renal LMS.

## Materials and Methods

### Data Extraction

Covering around 35% of the United States population, the SEER database contains data on cancer incidence and survival (14). We extracted data on cases of primary renal LMS and KIRC diagnosed from 1998 to 2016 using the SEER^*^Stat software released on August 8, 2019, version 8.3.6, from the “Incidence-SEER 18 Regs Custom Data (with additional treatment fields), Nov 2018 Sub (1975-2016 varying)” (http://www.seer.cancer.gov). Based on the third edition of the International Classification of Diseases for Oncology (ICD-O-3), our study included cases with the histology codes 8310/3 (clear cell adenocarcinoma) and 8890/3 (leiomyosarcoma). A total of 97,135 patients were identified after excluding 1,045 patients without a positive histology confirmation, 2,462 patients with non-renal primary carcinoma, 2,328 patients with 0 days or no survival time, and 15,759 patients with missing or unknown data of SEER cause-specific death classification information. Finally, 75,541 patients were enrolled, including 140 patients with primary renal LMS and 75,401 patients with KIRC.

### Clinicopathological Characteristics

The baseline clinicopathological characteristics included age, sex, race, marital status, laterality, tumor size, grade, and SEER stage. Combined with the most precise clinicopathological documentation, the tumor stage was divided into three categories based on the SEER program: localized (within organ), regional (extension to surrounding organs, adjacent tissues, or regional lymph nodes), and distant (direct extension or metastasis). The tumor grade was categorized as grade I (well-differentiated), grade II (moderately differentiated), grade III (poorly differentiated), and grade IV (undifferentiated). For cancer-specific survival (CSS), we defined deaths due to renal carcinoma as events and deaths caused by other reasons as censored observations (15). We excluded the patients without specific death classification information, as previously described. Treatment information including surgery, radiation, and chemotherapy status was also collected.

### Statistical Analysis

All analyses were conducted using the R software released in February 2020, version 3.6.3 (https://www.r-project.org/). The continuous variables were analyzed using *t*-tests, while the categorical variables were assessed using Pearson's chi-square or Fisher's exact tests using the “gmodels” package. Propensity score matching (PSM) was conducted using the “nonrandom” package to control the confounding factors between LMS and KIRC. The CSS and overall survival (OS) curves were estimated by Kaplan-Meier analyses and compared using a log-rank test in the “survival” and “survminer” packages. We also reported the corresponding hazard ratios (HRs) and 95% CIs. The risk factors for CSS and OS were estimated by the univariable and multivariable Cox proportional hazard regression models using the “survival” and “survminer” packages. We included and adjusted all the covariates with a univariable *p*-value ≤ 0.1 in the multivariable Cox proportional hazard regression model. Differences were considered statistically significant for two-sided *p* < 0.05.

## Results

### Clinicopathological Features of Patients With Primary Renal LMS and KIRC

In total, 140 patients with primary renal LMS and 75,401 patients with KIRC from 1998 to 2016 were finally enrolled. As summarized in Table 1, primary renal LMS and KIRC showed significant differences in sex, race, tumor size, tumor grade, SEER stage and number of patients receiving surgery, radiation, and chemotherapy. Compared with the KIRC group, the renal LMS group had more female patients (62.86 vs. 38%, *p* < 0.001), more non-white patients (25.71 vs. 14.12%, *p* < 0.001), larger tumor sizes (≥7 cm, 60 vs. 25.16%, *p* < 0.001), higher tumor grades (III–IV, 52.14 vs. 28.32%, *p* < 0.001), and higher SEER stages (regional and distant, 63.57 vs. 28.03%, *p* < 0.001). Fewer patients with renal LMS received surgery (82.14 vs. 94.31%, *p* < 0.001) whereas more received radiation (13.57 vs. 3.77%, *p* < 0.001) and chemotherapy (27.14 vs. 6.23%, *p* < 0.001).

**Table 1:** Clinicopathological characteristics of the patients with primary renal leiomyosarcoma and kidney renal clear cell carcinoma.

**Characteristics**	**Leiomyosarcoma**	**Clear cell carcinoma**	**P value**
	**No. (%) or Mean (± SD)**	**No. (%) or Mean (± SD)**	
**Age (years)**	59.39 ± 13.16	60.24 ± 12.42	0.44
**Sex**			<0.001
Female	88 (62.86)	28651 (38.00)	
Male	52 (37.14)	46750 (62.00)	
**Race**			<0.001
White	104 (74.29)	64207 (85.15)	
Black	21 (15.00)	5269 (6.99)	
Others^a^	15 (10.71)	5373 (7.13)	
Unknown	0 (0.00)	552 (0.73)	
**Marital status**			0.33
Married	84 (60.00)	47355 (62.80)	
Not married^b^	52 (37.14)	24685 (32.74)	
Unknown	4 (2.86)	3361 (4.46)	
**Laterality**			0.10
Left or Right	138 (98.57)	75122 (99.63)	
Bilateral	2 (1.43)	279 (0.37)	
**Tumor size (cm)**			<0.001
≤ 4	14 (10.00)	32692 (43.36)	
4~7	32 (22.86)	22348 (29.64)	
7~10	27 (19.29)	11661 (15.47)	
>10	57 (40.71)	7082 (9.39)	
Unknown	10 (7.14)	1618 (2.15)	
**Grade**			<0.001
Grade I	6 (4.29)	8448 (11.20)	
Grade II	21 (15.00)	33845 (44.89)	
Grade III	31 (22.14)	17234 (22.86)	
Grade IV	42 (30.00)	4119 (5.46)	
Unknown	40 (28.57)	11755 (15.59)	
**SEER stage**			<0.001
Localized	47 (33.57)	53797 (71.35)	
Regional	44 (31.43)	13156 (17.45)	
Distant	45 (32.14)	7976 (10.58)	
Unknown	4 (2.86)	472 (0.63)	
**Surgery**			<0.001
No	24 (17.14)	4165 (5.52)	
Yes	115 (82.14)	71107 (94.31)	
Unknown	1 (0.71)	129 (0.17)	
**Radiation**			<0.001
No/ Unknown	121 (86.43)	72560 (96.23)	
Yes	19 (13.57)	2841 (3.77)	
**Chemotherapy**			<0.001
No/Unknown	102 (72.86)	70705 (93.77)	
Yes	38 (27.14)	4696 (6.23)	

a *Others included American Indian/Alaskan native and Asian/Pacific islander*.

b *Not married included divorced, separated, single (never married), unmarried or domestic partner, and widowed*.

### Comparison of Survival Outcomes Between Primary Renal LMS and KIRC

**Figure 1:**
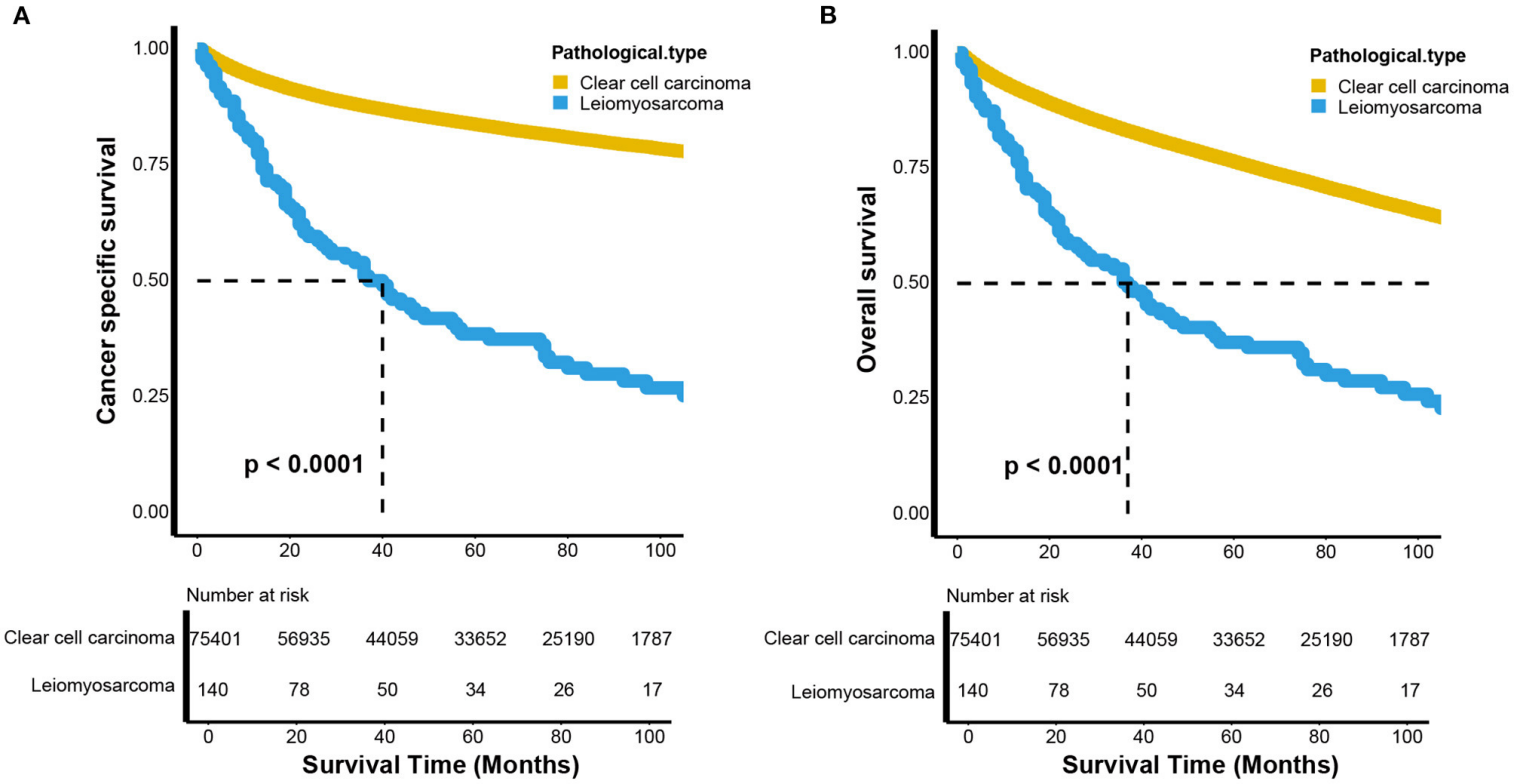
Kaplan–Meier estimates the **(A)** cancer-specific and **(B)** overall survival between renal leiomyosarcoma and renal clear cell carcinoma.

The CSS and OS curves were estimated by Kaplan-Meier analyses and compared using log-rank tests between renal LMS and KIRC. Both CSS (*p* < 0.001) and OS (*p* < 0.001) for renal LMS were significantly poorer than those for KIRC (Figure 1). For CSS, the median survival time of patients with renal LMS was 40 months (95% CI, 27–57 months), while the median survival time of KIRC was not reached. The 5-year CSS rates were 37.99 and 83.71%, respectively (Figure 1A). The median OS times of the patients with renal LMS and KIRC were 37 months (95% CI, 26–55 months) and 165 months (95% CI, 162–168 months), respectively. The 5-year OS rates were 36.66 and 76.31%, respectively (Figure 1B).

**Figure 2:**
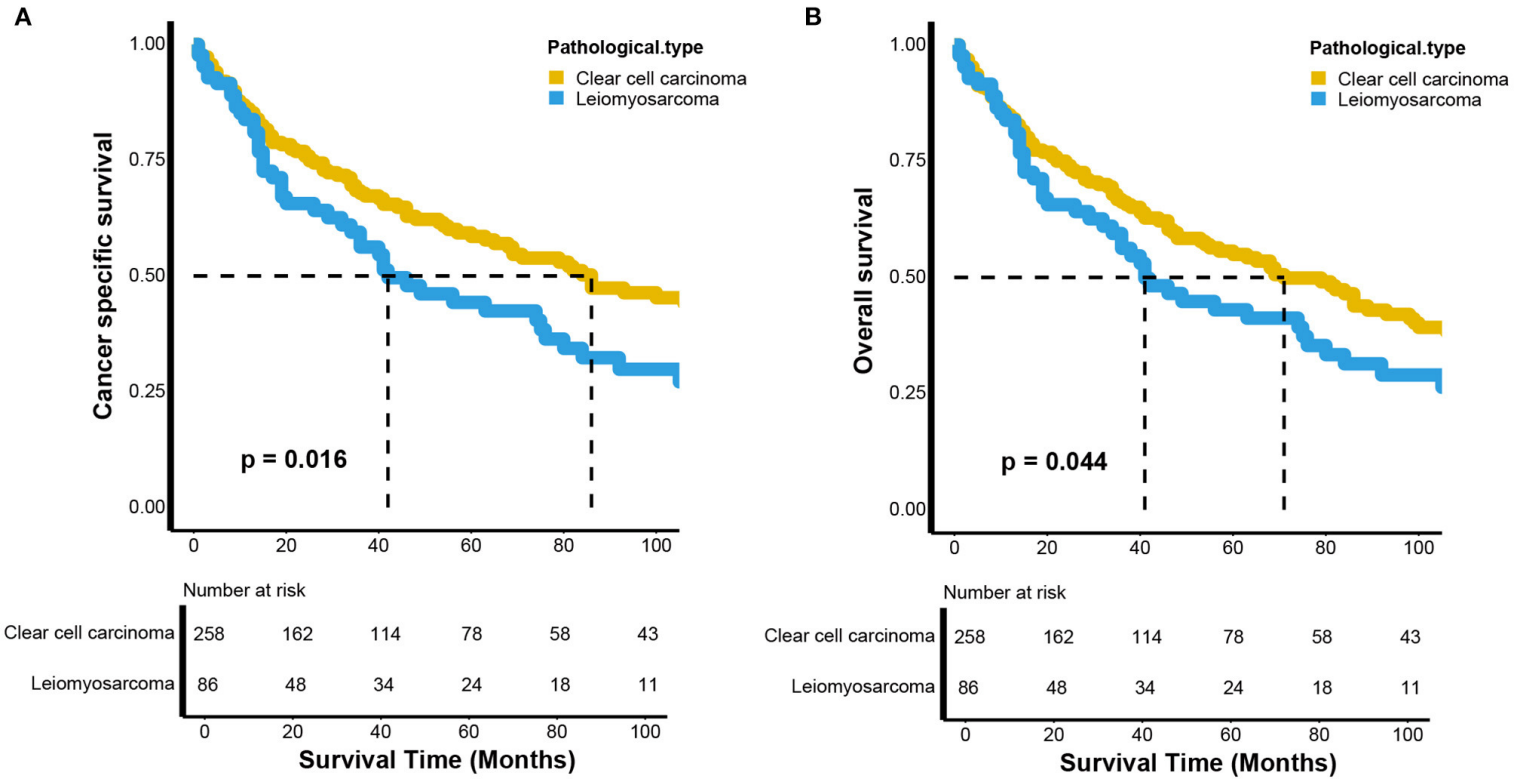
Kaplan–Meier estimates of the **(A)** cancer-specific and **(B)** overall survival between renal leiomyosarcoma and renal clear cell carcinoma after propensity score matching.

To control for the impact of the baseline characteristics on survival outcomes, a 1:3 (LMS vs. KIRC) PSM was conducted. Fifty-four patients with renal LMS were excluded due to missing baseline characteristics. Finally, 86 patients with renal LMS were included to match the KIRC patients. As shown in Table 1, there were no significant differences in baseline characteristics between the renal LMS and KIRC groups after matching. However, compared to those with KIRC, the patients with renal LMS still exhibited poor CSS (*p* = 0.016) and OS (*p* = 0.044) (Figure 2). For CSS, the median survival times of renal LMS and KIRC were 42 (95% CI, 34–80 months) and 86 months (95% CI, 68–148 months), respectively. The 5-year CSS rates were 43.37 and 58.54%, respectively (Figure 2A). For OS, the median survival time of renal LMS was 41 months (95% CI, 34–76 months), while that for KIRC was 71 months (95% CI, 55–98 months). The 5-year OS rates were 42.13 and 54.93%, respectively (Figure 2B).

### Prognostic Factors of CSS and OS in Patients With Primary Renal LMS

We first explored the potential prognostic factors of renal LMS using a univariable Cox proportional hazard regression model. As shown in Table 2, larger tumor sizes (>7 vs. ≤ 7 cm, *p* = 0.006, HR = 2, 95% CI, 1.23–3.26; *p* = 0.006, HR = 1.95, 95% CI, 1.22–3.13), higher tumor grades (Grades III and IV vs. Grades I and II, *p* < 0.001, HR = 4.29, 95% CI, 1.83–10.05; *p* = 0.002, HR = 3.12, 95% CI, 1.52–6.4), and higher SEER stages (regional vs. localized, *p* < 0.001, HR = 3.05, 95% CI, 1.67–5.57; *p* < 0.001, HR = 3.12, 95% CI, 1.74–5.61; distant vs. localized, *p* < 0.001, HR = 5.5, 95% CI, 2.97–10.19; *p* < 0.001, HR = 6.03, 95% CI, 3.32–10.94) were associated with poor CSS and OS, respectively. The patients receiving surgery (*p* < 0.001, HR =0.35, 95% CI, 0.2–0.59; *p* < 0.001, HR = 0.33, 95% CI, 0.2–0.55) and chemotherapy (*p* = 0.01, HR = 1.94, 95% CI, 1.21–3.09; *p* = 0.008, HR = 1.85, 95% CI, 1.17–2.91) were associated with favorable and poor CSS and OS, respectively.

**Table 2:** The univariable Cox proportional hazard regression model of cancer-specific and overall survival for primary renal leiomyosarcoma.

	**Cancer-special survival**		**Overall survival**	
**Characteristics**	**HR (95% CI)**	***P*-value**	**HR (95% CI)**	***P*-value**
Age	1.02 (1.00–1.04)	0.09	1.02 (1.00–1.04)	0.07
**Race**				
White	Reference	–	Reference	–
Black	1.23 (0.70–2.17)	0.47	1.17 (0.66–2.05)	0.60
Other^a^	1.19 (0.59–2.41)	0.63	1.23 (0.63–2.40)	0.54
**Sex**				
Female	Reference	–	Reference	–
Male	1.38 (0.90–2.14)	0.14	1.45 (0.96–2.18)	0.08
**Marital status**				
Married	Reference	–	Reference	–
Not married^b^	1.03 (0.66–1.59)	0.90	1.05 (0.69–1.59)	0.81
**Laterality**				
Left or right	Reference	–	Reference	–
Bilateral	0.56 (0.08–4.06)	0.57	1.01 (0.25–4.12)	0.99
**Tumor size**				
≤ 7	Reference	–	Reference	–
>7	2.00 (1.23–3.26)	0.006	1.95 (1.22–3.13)	0.006
**Grade**				
Grade I & II	Reference	–	Reference	–
Grade III & IV	4.29 (1.83–10.05)	<0.001	3.12 (1.52–6.40)	0.002
**SEER stage**				
Localized	Reference	–	Reference	–
Regional	3.05 (1.67–5.57)	<0.001	3.12 (1.74–5.61)	<0.001
Distant	5.50 (2.97–10.19)	<0.001	6.03 (3.32–10.94)	<0.001
**Surgery**				
No	Reference	–	Reference	–
Yes	0.35 (0.20–0.59)	<0.001	0.33 (0.20–0.55)	<0.001
**Radiation**				
None/unknown	Reference	–	Reference	–
Yes	0.78 (0.40–1.51)	0.46	0.97 (0.54–1.74)	0.91
**Chemotherapy**				
None/unknown	Reference	–	Reference	–
Yes	1.94 (1.21–3.09)	0.01	1.85 (1.17–2.91)	0.008

a *Others included American Indian/Alaskan native and Asian/Pacific islander*.

b *Not married included divorced, separated, single (never married), unmarried or domestic partner, and widowed*.

We then included and adjusted all the covariates with *p*-values < 0.1 in the multivariable Cox proportional hazard regression model. As shown in Table 3, higher tumor grades (Grades III and IV vs. Grades I and II, *p* = 0.002, HR = 6.04, 95% CI, 1.95–18.73; *p* = 0.005, HR = 3.78, 95% CI, 1.49–9.57) and higher SEER stages (regional vs. localized, *p* = 0.007, HR = 2.99, 95% CI, 1.35–6.6; *p* = 0.003, HR = 3.26, 95% CI, 1.51–7.02; distant vs. localized, *p* = 0.016, HR = 3.69, 95% CI, 1.27–10.68; *p* = 0.003, HR = 4.74, 95% CI, 1.7–13.17) remained independent risk factors for CSS and OS. However, only having undergone surgery was an independent protective factor for both CSS and OS (*p* = 0.03, HR = 0.26, 95% CI, 0.08–0.89; *p* = 0.04, HR =0. 28, 95% CI, 0.09–0.94).

**Table 3:** The multivariable Cox proportional hazard regression model of cancer-specific and overall survival for primary renal leiomyosarcoma.

	**Cancer-special survival**		**Overall survival**	
**Characteristics**	**HR (95% CI)**	***P*-value**	**HR (95% CI)**	***P*-value**
Age	1.01 (0.98–1.04)	0.42	1.02 (0.99–1.05)	0.31
**Sex**				
Female			Reference	–
Male			1.09 (0.60–1.96)	0.78
**Tumor size**				
≤ 7	Reference	–	Reference	–
>7	0.96 (0.45–2.03)	0.91	1.03 (0.49–2.16)	0.94
**Grade**
Grade I & II	Reference	–	Reference	–
Grade III & IV	6.04 (1.95–18.73)	0.002	3.78 (1.49–9.57)	0.005
**SEER stage**				
Localized	Reference	–	Reference	–
Regional	2.99 (1.35–6.60)	0.007	3.26 (1.51–7.02)	0.003
Distant	3.69 (1.27–10.68)	0.016	4.74 (1.70–13.17)	0.003
**Surgery**				
No	Reference	–	Reference	–
Yes	0.26 (0.08–0.89)	0.03	0.28 (0.09–0.94)	0.04
**Chemotherapy**				
None/unknown	Reference	–	Reference	–
Yes	1.07 (0.46–2.53)	0.87	0.97 (0.42–2.25)	0.95

### Subgroup Analyses of Survival Outcomes Between Patients Receiving and Not Receiving Surgery and Chemotherapy for Advanced Renal LMS

**Figure 3:**
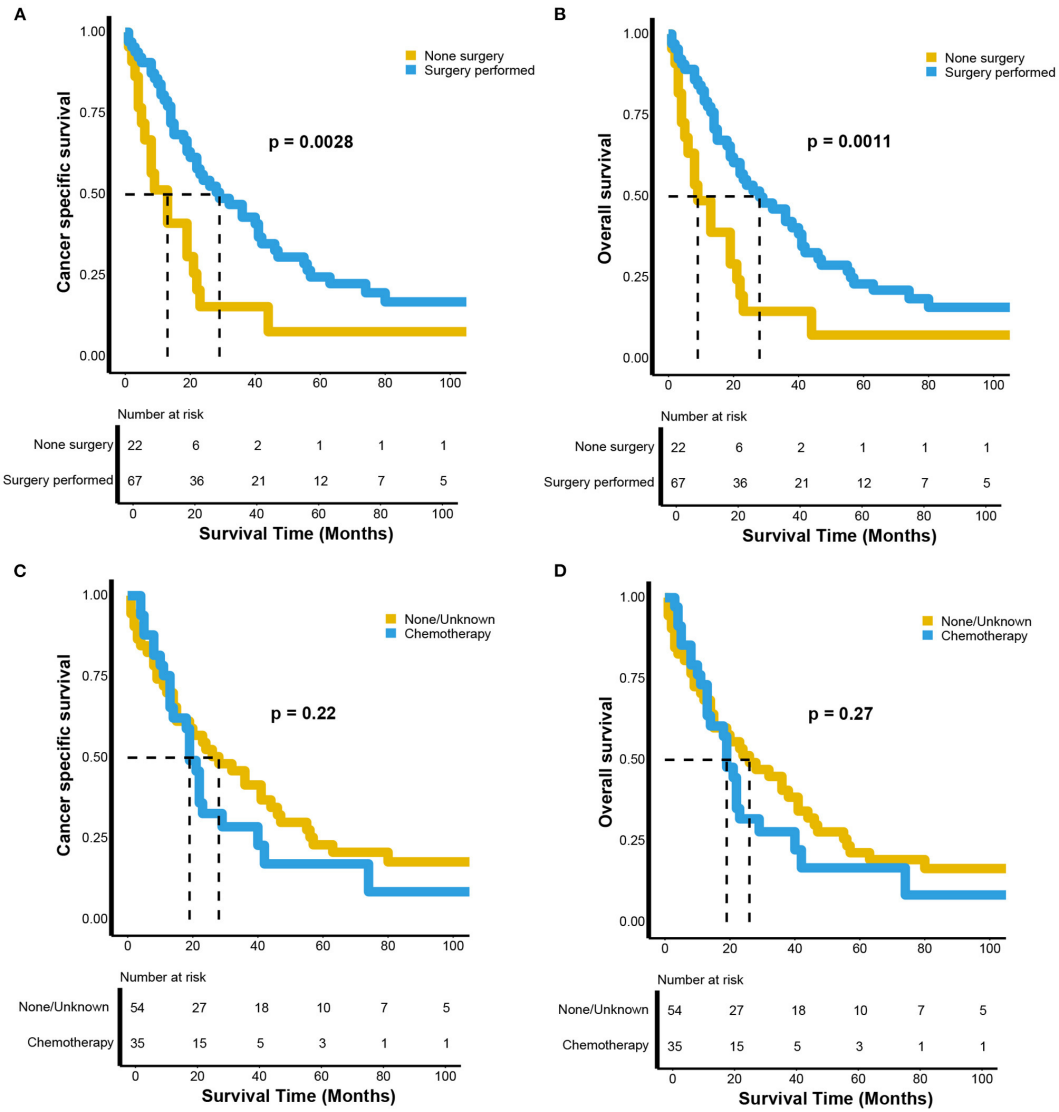
Kaplan–Meier estimates of the **(A)** cancer-specific and **(B)** overall survival between the patients with advanced renal leiomyosarcoma receiving surgery or not; the **(C)** cancer-specific and **(D)** overall survival between the patients with advanced renal leiomyosarcoma receiving chemotherapy or not.

We performed subgroup analyses of patients with advanced renal LMS, including those with regional and distant metastases. The CSS and OS curves were estimated using Kaplan-Meier analyses and compared using log-rank tests. Both the CSS (*p* = 0.003) and OS (*p* = 0.001) of patients who underwent surgery were significantly more favorable compared with those in patients who did not undergo surgery (Figures 3A,B). For CSS, the median survival times were 29 (95% CI, 22–46 months) and 13 months (95% CI, 6–22 months) for patients who did and did not undergo surgery, respectively. The 5-year CSS rates were 22.53 and 7.71%, respectively (Figure 3A). For OS, the median survival times were 28 (95% CI, 20–41 months) and 9 months (95% CI, 6–22 months) for patients who did and did not undergo surgery, respectively. The 5-year OS rates were 21.14 and 7.31%, respectively (Figure 3B). However, there were no survival differences between patients who received chemotherapy and those who did not both for CSS (*p* = 0.22) and OS (*p* = 0.27). For CSS, the median survival times were 19 (95% CI, 14–40 months) and 28 months (95% CI, 15–46 months) for patients who did and did not receive chemotherapy, respectively. The 5-year CSS rates were 17.19 and 20.78%, respectively (Figure 3C). For OS, the median survival times were 19 (95% CI, 13–29 months) and 26 months (95% CI, 15–44 months) for patients who did and did not receive chemotherapy, respectively. The 5-year OS rates were 16.7 and 20.31%, respectively (Figure 3D).

## Discussion

Primary renal LMS remains an exceedingly rare entity, although it is the most common pathological subtype of renal sarcoma, accounting for 50–60% of all cases (4). Its clinicopathological characteristics, potential treatment choice, and survival outcomes have not been fully characterized. We studied the largest cohort of 140 cases of primary renal LMS and summarized its clinicopathological characteristics, treatment, and survival outcomes and systematically compared these with those for KIRC. There were significant differences in sex, race, tumor size, grade, SEER stage, surgery status, radiation, and chemotherapy between patients with renal LMS and KIRC. The renal LMS group also exhibited poorer CSS and OS than the KIRC group before and after PSM. Univariate and multivariate Cox proportional hazard regression models were used to identify potential prognostic factors. We found that higher tumor grades and SEER stages were independent risk factors while having undergone surgery was an independent protective factor. While receiving chemotherapy was a risk factor in the univariate Cox proportional hazard regression model, its prognostic impact disappeared in the multivariate analysis. We wondered if there were confounding factors related to this outcome, especially the tumor stage. Therefore, we performed a subgroup analysis of patients with advanced renal LMS, including those with regional and distant metastases. We found that these patients benefited from surgery and that there was no survival difference between the patients with advanced renal LMS who received chemotherapy and those who did not.

Advanced renal LMS is reportedly more common in female patients, and there is an increasing incidence in older patients (16, 17). However, studies have also reported an equal distribution between male and female patients and a mean age at diagnosis of ~60 years (4, 9). In our study, the mean age at diagnosis was 59.39 years. We also observed that tumors were more common in female patients (62.86%). Although inconclusive, this result may be related to hormones (18). Unlike the low malignant potential and relatively favorable prognosis of KIRC, renal LMS possesses rapid growth characteristics and is always diagnosed in the late stage (19). This highly aggressive tumor generally arises from the renal pelvis, capsule, or vein (19). As LMS originates from the mesenchymal components and lacks natural barriers, primary renal LMS can grow large sizes (20). Moreover, the highly distensible potential of the retroperitoneum provides space for tumor growth (14). Consistent with these previous observations, we also observed that renal LMS presented a larger tumor size, higher tumor grade, and higher tumor stage than KIRC. Therefore, it is not surprising that renal LMS showed an extremely poor prognosis in previous and present studies (8–10). Even after PSM, renal LMS still exhibited poorer CSS and OS than KIRC in our study. However, other studies have reported relatively favorable survival outcomes for renal LMS (6, 21) compared with KIRC (7). This may be due to the relatively smaller number of patients in other studies. To our knowledge, this is the largest report on primary renal LMS.

Because of its rarity, the treatment choices are also controversial. Radical nephrectomy is widely considered the gold treatment choice for renal LMS (3, 6, 20). In cases with a negative surgical margin, the 5-year survival rate can reach 60% (16). While a study reported nephron-sparing surgery for renal LMS (6), the reported renal mass of the patient remained unchanged for 3 years. In our study, we found that surgery was a protective factor in both the univariate and multivariate Cox proportional hazard regression models. In addition, our subgroup analysis indicated that patients with advanced renal LMS still benefited from surgery. As renal LMS always appears with large tumor size and at a late stage, most patients are not suitable for nephron-sparing surgery. To achieve better oncologic control, radical nephrectomy could be the gold treatment option (3, 6). While some cases have reported that renal LMS could benefit from chemotherapy or radiation (11, 12, 22), others reported that chemotherapy or radiation had no benefit (13). We found that the patients did not benefit from radiation. A large multinational clinical trial comparing radiotherapy plus surgery with surgery alone in retroperitoneal sarcoma concluded that radiation should not be recommended for sarcoma (23). While receiving chemotherapy was a risk factor in the univariate Cox proportional hazard regression model, its prognostic impact disappeared in the multivariate analysis. We speculated if there were confounding factors related to this outcome, especially the tumor stage. Our subgroup analysis revealed no survival difference between the patients with advanced renal LMS receiving or not receiving chemotherapy. Patients with late tumor stages were more likely to receive chemotherapy, which could explain why receiving chemotherapy was a risk factor in the univariate Cox proportional hazard regression model. In metastatic uterine LMS, docetaxel combined with gemcitabine is an effective treatment option (24). However, a multinational, randomized trial comparing the observations with adjuvant chemotherapy closed because of a failure to recruit patients. Therefore, whether early-stage uterine LMS can benefit from adjuvant docetaxel plus gemcitabine remains unknown (24). As some renal LMS cases are present in metastatic or bilateral diseases (25), radical nephrectomy may not be suitable in such cases; thus, chemotherapy is the only palliative treatment choice.

Our study has some limitations. First, our data were extracted from the SEER database and could not overcome the inherent limitations of the retrospective design. Second, the SEER data lack details on chemotherapy and radiation type and duration. Therefore, we could only categorize the patients as receiving chemotherapy/radiation or “none/unknown,” which could generate a bias. Third, we included a relatively small number of patients, which could make the results less convincing. However, to our knowledge, this is the largest study on primary renal LMS to date.

## Conclusion

Primary renal LMS is an exceedingly rare entity with distinct clinicopathological features and a poor prognosis. A higher tumor grade and late stage may indicate a poor prognosis. Complete tumor resection remains the first treatment choice, and chemotherapy could be a palliative treatment choice for advanced patients. Long-term and large population-based studies are needed to confirm these findings.

## Data Availability Statement

Publicly available datasets were analyzed in this study. This data can be found here: https://seer.cancer.gov/.

## Author Contributions

CC and WW: conception and design. WW: administrative support. XJ, FX, and XC: provision of study materials or patients. CC, XJ, FX, and XC: collection and assembly of data. CC and XJ: data analysis and interpretation. All authors manuscript writing and final approval of manuscript.

## Conflict of Interest

The authors declare that the research was conducted in the absence of any commercial or financial relationships that could be construed as a potential conflict of interest.

## Publisher's Note

All claims expressed in this article are solely those of the authors and do not necessarily represent those of their affiliated organizations, or those of the publisher, the editors and the reviewers. Any product that may be evaluated in this article, or claim that may be made by its manufacturer, is not guaranteed or endorsed by the publisher.

## Supplementary Material

Table S1 Clinicopathological characteristics of patients with primary renal leiomyosarcoma and kidney renal clear cell carcinoma after propensity score matching. aOthers included American Indian/Alaskan native and Asian/Pacific Islander. bNot married included divorced, separated, single (never married), unmarried or domestic partner and widowed.

## Abbreviations

LMS: leiomyosarcoma
SEER, the Surveillance, Epidemiology: and End Results
KIRC: renal clear cell carcinoma
PSM: propensity score matching
CSS: cancer specific survival
OS: overall survival
HRs: hazard ratios
CI: confidence intervals.
