# Supplementary material for: Clinicopathological Characteristics and Survival Outcomes of Primary Renal Leiomyosarcoma
Source: Front Surg. 2021 Oct 21;8:704221. doi: 10.3389/fsurg.2021.704221 (PMC8566676; doi:10.3389/fsurg.2021.704221)
Supplement: Table S1 — Clinicopathological characteristics of patients with primary renal leiomyosarcoma and kidney renal clear cell carcinoma after propensity score matching. aOthers included American Indian/Alaskan native and Asian/Pacific Islander. bNot married included divorced, separated, single (never married), unmarried or domestic partner and widowed. [file Table_1.docx]

| Characteristics | Leiomyosarcoma | Clear cell carcinoma | P value |
| --- | --- | --- | --- |
|  | No. (%) or Mean (± SD) | No. (%) or Mean (± SD) |  |
| **Age (years)** | 59.64 ± 12.65 | 59.24 ± 11.94 | 0.80 |
| **Sex** |  |  | 0.90 |
| Female | 54 (62.79) | 164 (63.57) |  |
| Male | 32 (37.21) | 94 (36.43) |  |
| **Race** |  |  | 0.97 |
| White | 65 (75.58) | 198 (76.74) |  |
| Black | 10 (11.63) | 29 (11.24) |  |
| Others^a^ | 11 (12.79) | 31 (12.02) |  |
| **Marital status** |  |  | 0.85 |
| Married | 53 (61.63) | 162 (62.79) |  |
| Not married^b^ | 33 (38.37) | 96 (37.21) |  |
| **Laterality** |  |  | 0.44 |
| Left or Right | 85 (98.84) | 257 (99.61) |  |
| Bilateral | 1 (1.16) | 1 (0.39) |  |
| **Tumor size (cm)** |  |  | 0.96 |
| ≤4 | 10 (11.63) | 30 (11.63) |  |
| 4~7 | 20 (23.26) | 66 (25.58) |  |
| 7~10 | 16 (18.60) | 43 (16.67) |  |
| >10 | 40 (46.51) | 119 (46.12) |  |
| **Grade** |  |  | 0.96 |
| Grade I | 6 (6.98) | 17 (6.59) |  |
| Grade II | 16 (18.60) | 49 (18.99) |  |
| Grade III | 27 (31.40) | 74 (28.68) |  |
| Grade IV | 37 (43.02) | 118 (45.74) |  |
| **SEER stage** |  |  | 0.81 |
| Localized | 33 (38.37) | 99 (38.37) |  |
| Regional | 33 (38.37) | 91 (35.27) |  |
| Distant | 20 (23.26) | 68 (26.36) |  |
| **Surgery** |  |  | 0.65 |
| No | 6 (6.98) | 22 (8.53) |  |
| Yes | 80 (93.02) | 236 (91.47) |  |
| **Radiation** |  |  | 1.00 |
| No/ Unknown | 77 (89.53) | 231 (89.53) |  |
| Yes | 9 (10.47) | 27 (10.47) |  |
| **Chemotherapy** |  |  | 0.52 |
| No/Unknown | 68 (79.07) | 212 (82.17) |  |
| Yes | 18 (20.93) | 46 (17.83) |  |
